# Scale Selection and Machine Learning-based Cell Segmentation and Tracking in Time Lapse Microscopy

**DOI:** 10.21203/rs.3.rs-5228158/v1

**Published:** 2024-10-30

**Authors:** Nagasoujanya Annasamudram, Jian Zhao, Aashish Prashanth, Sokratis Makrogiannis

**Affiliations:** 1 Division of Physics, Engineering, Mathematics and Computer Science, Delaware State University, Dover, DE 19901, USA

**Keywords:** cell tracking, time-lapse series, spatio-temporal features, automated scale selection, neural net

## Abstract

Monitoring and tracking of cell motion is a key component for understanding disease mechanisms and evaluating the effects of treatments. Time-lapse optical microscopy has been commonly employed for studying cell cycle phases. However, usual manual cell tracking is very time consuming and has poor reproducibility. Automated cell tracking techniques are challenged by variability of cell region intensity distributions and resolution limitations. In this work, we introduce a comprehensive cell segmentation and tracking methodology. A key contribution of this work is that it employs multi-scale space-time interest point detection and characterization for automatic scale selection and cell segmentation. Another contribution is the use of a neural network with class prototype balancing for detection of cell regions. This work also offers a structured mathematical framework that uses graphs for track generation and cell event detection. We evaluated cell segmentation, detection, and tracking performance of our method on time-lapse sequences of the Cell Tracking Challenge (CTC). We also compared our technique to top performing techniques from CTC. Performance evaluation results indicate that the proposed methodology is competitive with these techniques, and that it generalizes very well to diverse cell types and sizes, and multiple imaging techniques.

## Introduction

Monitoring and tracking of cell motion is a key component for understanding disease mechanisms and evaluating treatments ^1,2^. Cell migration, i.e., cell motion that is accompanied by morphological changes, and mitosis, i.e., cell division into two daughters, occur during development, wound healing, and immune responses. In cancer, for example, cell migration is critical in almost all stages of metastasis. Quantifying the progression of cell cycles enables us to understand the effect of drug treatments. In addition, tissue engineering and stem cell manufacturing require quantification of stem cell proliferation in vitro ^3–5^.

Time-lapse optical microscopy techniques have been widely used for studying cell migration. The most common techniques toward this end are fluorescence microscopy, phase contrast ( PhC ), and differential intereference contrast (DIC). These technologies produce image sequences frequently in the order of terabytes with a large number of frames, and dense or sparse cell environments depending on the assay. Many labs use manual cell tracking techniques. Such techniques require a significant amount of time and are characterized by poor reproducibility. Hence, there is a clear need for the development of automated, reproducible cell segmentation and tracking methods.

Automated cell tracking mainly entails finding and segmenting all cell occurrences in a time-lapse sequence and defining temporal relationships. This task is complicated by the following factors: (1) variability of cell shapes, sizes, and intensity distributions, (2) variability of cell contrast produced by different microscopy techniques, (3) temporal and spatial resolution limitations, and varying contrast to noise ratio even within a frame of the sequence. Therefore the development of generalizable computer-based tools for cell tracking is a popular open problem in the research community ^5–8^. Recent software tools implement segmentation and tracking stages ^9–11^ but were mostly developed for specific microscopy techniques such as fluorescence ^10^, or phase contrast microscopy ^11^. Due to the significance and popularity of the cell tracking problem, computer-based cell segmentation and tracking competitions, such as the Cell Tracking Challenge (CTC) ^6,12^, have emerged to provide a common basis for performance comparisons. Furthermore, numerous techniques have been published to tackle cell segmentation, detection and tracking ^9,13–21^.

In this work, we propose a methodology for generalizable cell segmentation and tracking that can be applied to multiple types of cells and microscopy imaging techniques. A frequent pitfall in segmentation of microscopy sequences is the unpredictable change of levels of contrast and noise between different sequences and even from frame-to-frame within a sequence. To address this key issue, we aim to identify the optimal scale for cell detection and segmentation using multi-scale space-time interest point detectors. Analysis of space, time, and space-time interest points in a multi-scale hierarchical strategy has yielded promising results for multiple computer vision tasks, such as segmentation and object detection ^22–28^. Here we propose to use these detectors (i) to select an optimal scale for each frame, (ii) to identify interest points on the cells, and (iii) to guide segmentation of cell boundaries. We also introduce a multi-layer neural network to detect the cell regions and complete the cell segmentation stage. Cell detection classes are frequently imbalanced, as there are a lot more noisy background regions than cell regions. This imbalance introduces classification bias. To address this issue, we propose to use the method of Density-Based Spatial Clustering of Applications with Noise (DBSCAN) ^29–31^ before training, to identify the main cluster prototypes in the feature space and resample the training samples. DBSCAN is an unsupervised nonparametric technique that has also been previously proposed for 3D cell separation ^9^, and in a U-Net boosting training strategy for brain tumor segmentation in MRI^32^. In the cell tracking stage, we use a graph-based technique to identify cell migration and cell mitosis events. First, we employ optical flow-based motion compensation and cell matching. Next, we form cell tracklets by linking cell instances across frames, and we identify cell migration and mitosis events to complete the tracking stage.

A key contribution of the proposed technique is that it incorporates the concept of space-time iterest point detection and characterization into automatic scale selection, segmentation, and scale detection. An additional strength is the use of a machine learning technique with class prototype balancing for cell detection. Furthermore, our method offers a well-defined and structured mathematical framework that uses graphs for track generation and cell event detection. We evaluated the segmentation, detection and tracking performance of our method on four fluorescence and two phase contrast time-lapse sequences of the Cell Tracking Challenge. In addition, we compared our technique to top performing techniques from CTC. The performance evaluation results indicate that the proposed methodology is competitive with these techniques, and it generalizes very well to diverse cell types and sizes, and diverse spatial and temporal resolutions.

## Methods

In this section we detail the two main components of our framework: cell segmentation and detection, and cell tracking.

### Cell Segmentation and Detection

**Figure 1.**
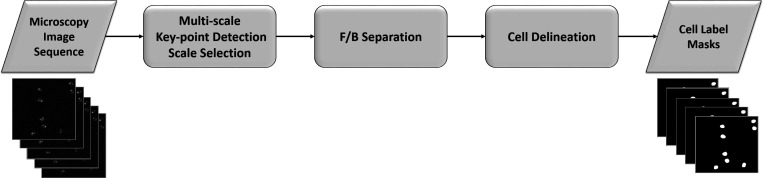
The main stages of cell segmentation and detection. Key processes are multi-scale interest point detection for automated scale selection and segmentation, and neural network classification for cell detection.

We display the main stages for cell segmentation and detection in Figure 1. We describe each of these stages next, with emphasis on multi-scale interest point detection for automated scale selection and segmentation, and neural network-based foreground/background separation for cell detection.

#### Multi-scale Cell Interest Point Detection and Segmentation

##### Spatio-temporal Anisotropic Diffusion

This filter is applied by solving a system of three coupled PDEs, each resembling the diffusion equation with anisotropic diffusion ^33,34^. This system is solved for each frame and its direct temporal neighbors, denoted by τ={t−1,t,t+1}:

(1) $$\frac{\partial L(i,j,\tau ,s)}{\partial s}=g\left(\left|\nabla L\left(i,j,\tau ,s\right)\right|\right)\cdot \Delta L\left(i,j,\tau ,s\right)+\nabla g\left(\left|\nabla L\left(i,j,\tau ,s\right)\right|\right)\cdot \nabla L\left(i,j,\tau ,s\right).$$

Here, i and j are the spatial indices for the pixels. τ is frame index, s is the scale index. This stage generates multi-scale diffused frame maps. The diffused frame maps are utilized for spatio-temporal feature detection, segmentation, and foreground/background separation.

##### Spatio-temporal Interest Point (Feature) Detectors

The goal is to detect corners, blob structures and other feature types in the spatio-temporal domain ^22,27^. Earlier techniques have used Laplacian-of-Gaussian blob detectors in a single scale to detect and count cell nuclei. ^35,36^. We expect that the use of non-linear diffusion models will enable the detection of non-spherical features that may correspond to non-circular cell shapes and how they evolve in multiple scales. We also estimate motion displacement vector fields of the moving regions ^37–40^ and add them to the feature set for machine learning-based cell detection.

###### Spatio-temporal Moments:

These are the spatio-temporal second moments ^22^ computed as an extension of Harris corner detector. This approach computes the structure tensor

(2) $$M=w\left(\cdot ,\sigma_{i}^{2},\tau_{i}^{2}\right)*\left(\begin{array}{ccc} L_{x}^{2} & L_{x}L_{y} & L_{x}L_{z}\\ L_{y}L_{x} & L_{y}^{2} & L_{y}L_{z}\\ L_{z}L_{x} & L_{z}L_{y} & L_{z}^{2} \end{array}\right),$$

where $w\left(\cdot ,\sigma_{i}^{2},\tau_{i}^{2}\right)$ denotes a smoothing kernel with integrating factors $\sigma_{i}^{2},\tau_{i}^{2}$. Then, it uses the Harris corner detection criterion S to identify the points of interest

(3) $$S=det(M)-\kappa \cdot trace^{3}(M).$$

###### Spatio-temporal Hessian:

This approach computes the Hessian matrix H in the space-time domain

(4) $$H\left(\cdot ,\sigma^{2},\tau^{2}\right)=\left(\begin{array}{lll} L_{xx} & L_{xy} & L_{xz}\\ L_{yx} & L_{yy} & L_{yz}\\ L_{zx} & L_{zy} & L_{zz} \end{array}\right).$$

It measures the strength of the interest points using the determinant magnitude, S=|det(H)|.

###### Spatial Hessian on Temporal Derivative:

This approach computes the spatial Hessian matrix on first-order temporal derivatives $L_{t}$ that we denoted by $H_{t}$. It measures the strength the interest points using the determinant S=|det(H)|.

###### Motion Map Computation and Cell Deformation

We estimate the motion of each cell in the prior frame by the Combined Local/Global Optical Flow (CLGOF) technique ^37-40^. We use the computed motion vector field as a region descriptor for cell detection, and to warp the cells of each prior frame onto the cells of each processed frame.

##### Automatic Scale Selection in Spatio-temporal Domain

**Figure 2.**
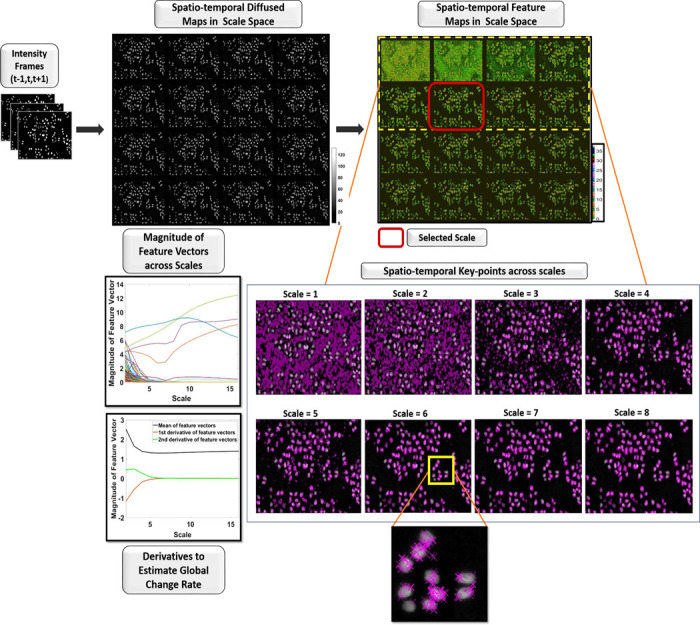
Example of scale generation and automatic selection. It shows the generation of spatio-temporal non-linear scale space, computation of feature map, and the analysis of interest points (features) over the generated scales for automated scale selection.

In this stage, we aim to identify the image scale that preserves meaningful cell visual content while suppressing the noise. For each frame, we develop a stack of multi-scale feature descriptors in the spatio-temporal domain. The feature loci are defined by regional maxima on each scale. We identify the optimal scale as the one that minimizes the number of noisy features that should diminish rapidly in the initial scales, while preserving the features of cells that may sustain longer along the scale space continuum. To this end, we analyze the second order derivatives of features across multiple scales similarly to approaches applied to natural video sequences ^27^. First, we normalize the feature vectors to be able to automatically choose the diffused map with best feature descriptors. Next, we compute the second-order derivative of the spatio-temporal feature vectors to determine a decision criterion for scale selection. Then, the selected scale diffused map along with the regional maxima drive the subsequent cell segmentation and detection stages. Figure 2 illustrates an example of scale generation, keypoint computation, and scale selection.

##### Watershed and Interest Point-based Segmentation

This stage generates markers to drive the watershed segmentation by fusing the regional interest point feature maxima with the regional minima of the edge map computed on the selected diffused map. First, we invert the stochastic map produced by Parzen density estimation to form regions separated by spatio-temporal discontinuities. Then, we fuse the feature maxima with the density map and produce watershed superpixels.

#### Machine Learning-based Cell Detection

Here we introduce a model that can classify the watershed superpixels into cells (foreground) and non-cell (background) classes. We employed a fully connected neural network to perform this classification. We train the network on static and dynamic descriptors formed by the regional averages and variances of diffused map intensities, spatio-temporal descriptors, motion displacement vector magnitudes, spatio-temporal intensity gradient magnitudes, and regional areas. The training vectors are designed to enable the model to learn foreground pixels for cells with irregular shapes, variable feature magnitudes and rates of motion, and varying contrast to noise ratios.

One difficulty in effective training of this machine learning model is the class imbalance between cell and non-cell regions as the class of non-cells typically has many more samples than the cell class. This imbalance frequently leads to classification bias. In this work, we propose to use DBSCAN (Density-Based Spatial Clustering of Applications with Noise) ^29–31^ to identify the main cluster prototypes formed in the feature space by the training samples. We measure the cluster sizes (or cardinalities) and perform balancing of the cluster sizes by undersampling the cluster with the most elements. This unsupervised learning stage balances class prototypes to improve neural network training.

The main contributions of DBSCAN are cluster modeling and a clustering algorithm. It is based on the assumption that clusters correspond to areas of high density that are separated by areas of lower density. The data points are classified into core points, border points, and noise. It performs nonparametric density estimation by computing the number of points within the ε-neighborhood of each query point. DBSCAN effectively computes the transitive closure of an equivalence relation. It can identify clusters of arbitrary shapes and sizes, while being less sensitive to noise and outliers.

To balance the cluster cardinalities in the feature space, we under-sample the most populous cluster to equalize its size with the size of the second most populous cluster. This operation reduces class imbalance in the training data without changing the structure of the data in the feature space. In DBSCAN we used Mahalanobis distances for improved modeling of arbitrary shapes of clusters.

Next, we train the neural network model on the balanced dataset produced by the previous step. Then, employed a network with layer structure of 9×12×6×2 nodes. The fully connected layers are followed by RELU activation. The first layer takes as input the regional feature vectors. The network predicts if the input region descriptor corresponds to a cell, or a non-cell region. We employ the cross entropy loss function for training.

#### Cell Boundary Refinement and Cell Cluster Separation

In this stage, we aim to refine the cell boundaries detected from the previous stage of foreground/background separation. We employ a region-based energy minimizing level-set model with a local clustering criterion to delineate the cell region. Next, we identify and separate adherent (or clustered) cells. We compute the solidity to identify the clustered cells, and then we compute the signed distance map of the identified regions. The watershed lines of the distance map determine the boundaries that divide the cells.

### Cell Tracking

**Figure 3.**
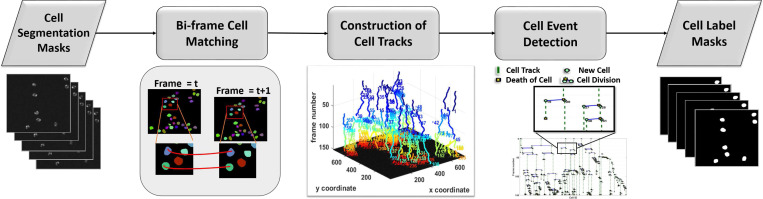
Cell tracking method overview. Bi-frame cell matching is applied first by motion compensation and max likelihood matching. Cell tracklets are formed by linking cell correspondences over the complete time sequence. A graph structure is used to store the cell relationships and detect migration and mitosis events.

A cell tracking technique should effectively manage key cell events in a sequence, such as cell migration (cell motion that is accompanied by morphological changes), cell mitosis (division into two daughters), cell disappearance (leaving the field of view or collision), cell apoptosis/necrosis (cell death), new cells entering the field of view, and cell reappearance (re-entering the field of view). This is a challenging task mainly because of low temporal resolution and variable types of cell motion. For example, the biological cells may follow a Brownian movement, which makes the motion estimation very hard. We developed a graph optimization technique that can be divided into the following stages: bi-frame cell matching, construction of cell tracks and cell event detection ^41^ as shown in Figure 3.

#### Bi-frame Cell Matching

In this stage we find cell correspondences between two consecutive frames that we denote by current frame and prior frame. The problem of finding the association with the highest probability is performed by determining the max-likelihood matching for each cell of the current frame among all the warped cells of the prior frame by the motion field that was found by CLGOF as described before.

Let $X_{t}=\left\{x_{1}^{t},x_{2}^{t},\ldots ,x_{n}^{t}\right\}$ and $\Xi_{t}=\left\{\xi_{1}^{t},\xi_{2}^{t},\ldots ,\xi_{n}^{t}\right\}$ be the set of observations and the set of states, respectively, for $n_{t}$ cells at time t. We assume a Markov process such that the transition probability depends only on the state of the mother cell or the same cell in previous frame $P\left(S_{t+1}|S_{t}\right)$. We compute the likelihood $\pi_{ij}^{t+1}$ that the cell i in frame t+1 is connected with cell j in frame t based on the spatial proximity between cells of warped indicator function $\overset{ˆ}{L}(\omega +\overset{ˆ}{\delta },t+1)$ and cells of the current indicator function (ω,t):

(5) $$\hat{j}=arg\;\underset{j}{\max}\pi_{ij}^{t+1}=arg\;\underset{j}{\max}p\left(x_{i}^{t+1}|x_{j}^{t},\xi_{j}^{t}\right).$$

We also use a reject option that signifies cell appearance when the maximum likelihood is still very low. After making a decision we assign the predicted cell class $\overset{ˆ}{j}$ to the cell indicator map.

#### Construction of Cell Tracks

The cell associations are stored in a structure of linked lists. To form the cell tracks, we traverse the linked lists from the last frame to the first. The identified maximum likelihood tracks form a forest of minimal cost chains.

We use the created linked lists to construct the cell tracks. We traverse the set of cell states in reverse chronological order and we store the cell track information in a structure with elements $Q=\left[t,\xi_{m}^{t},t_{p},\xi_{n}^{t_{p}}\right]$, that contain the frame id t, cell label (indicator) id $\xi_{m}^{t}$, previous frame id $t_{p}$, and previous label id $\xi_{n}^{t_{p}}$.

This stage produces a cell track structure that holds the tracks $\phi_{i}$ and addresses cell appearance and disappearance. However, some tracks may be partially overlapping in cell division occurrences, where different cells have a common ancestor. We address these cases by identifying the overlapping parts of two tracks $\phi_{j}$ and $\phi_{k}$, and creating three new tracks, one for the mother $\phi_{p}=\phi_{j}\cap \phi_{k}$, and the 2 daughters $\phi_{q}=\phi_{j}-\left(\phi_{j}\cap \phi_{k}\right)$ and $\phi_{r}=\phi_{k}-\left(\phi_{j}\cap \phi_{k}\right)$.

#### Cell Event Detection

Cell tracking results can be represented using an acyclic oriented graph G=(V,E) that consists of a vertex set V and an edge set E such that E⊂V×V. The graph vertices represent the detected cells and its edges represent temporal relations between the cells. The condition for an ordered vertex pair $E_{i,j}^{t_{1},t_{2}}\left(\xi_{i}^{t_{1}},\xi_{j}^{t_{2}}\right)$ to belong to the edge set E is expressed as:

$$\left(i=j\wedge t_{2}=t_{1}+1\right)\vee \left(i\neq j\wedge t_{1}<t_{2}\wedge P\left(\xi_{j}^{t_{2}}\right)=\xi_{i}^{t_{1}}\right)\Rightarrow E_{i,j}^{t_{1},t_{2}}\left(\xi_{i}^{t_{1}},\xi_{j}^{t_{2}}\right)\in E.$$

The function P:Ξ→Ξ, where Ξ is the universe of cell states $\xi_{i}$, returns the mother $\xi_{m}$ of an entity $\xi_{i}$ that is $\xi_{m}=P\left(\xi_{i}\right)$. The first condition in the previous expression represents cell migration, while the second condition represents cell division. We assign graph node labels $L_{G}(V)$ to cells in each indicator image that determines a global indicator function $F_{L}:\Omega \to L_{G}$.

## Experiments and Results

In our performance evaluation experiments, we used Cell Tracking Challenge (CTC) datasets ^12^. CTC datasets consist of time-lapse microscopy sequences and annotations that can serve as benchmarks for cell segmentation, detection and tracking. Our experiments can be divided into two main parts. In the first experiment, we evaluated the performance of our method on CTC training sequences. In the second experiment, we evaluated the performance of our technique in comparison to results of other published techniques that participated in CTC and produced results on the same sequences. Next, we describe the CTC datasets, the evaluation measures, and the results for each experiment.

### Datasets

We utilized 4 datasets of fluorescence (Fluo) microscopy and 2 phase-contrast (PhC) microscopy for training and testing. Each dataset contains two sequences labeled 01 and 02, along with two sets of reference masks named gold standard corpus or gold-truth (GT) and silver standard corpus or silver truth (ST).

We evaluated our method on fluorescence microscopy sequences of low contrast cytoplasm of rat mesenchymal stem cells Fluo-C2DL-MSC (MSC), low contrast nuclei of cervical cancer cells Fluo-N2DL-HeLa (HeLa), high contrast nuclei of mouse embryonic stem cells Fluo-N2DH-GOWT1 (GOWT1) and high contrast simulated nuclei of human leukemia cells (HL-60) Fluo-N2DH-SIM+ (SIM+) from fluorescence microscopy. The phase contrast microscopy datasets include high contrast cytoplasm of glioblastoma-astrocytoma U373 cells PhC-C2DH-U373 (U373) and low contrast cytoplasm of pancreatic stem cells PhC-C2DL-PSC (PSC). The datasets vary in noise levels, cell densities, numbers of cells leaving and entering the field of view, resolution, and numbers of mitotic events.

The reference label set consists of the GT reference datasets that are manually annotated by experts with background in biology at three different institutions, and the ST reference datasets that contain computer-generated annotations from the top results submitted by former participants.

### Performance Evaluation Measures

The CTC training dataset serves as a validation set for measuring segmentation accuracy by the Dice Similarity Coefficient (DSC), Jaccard index, SEG measure (SEG), and DET measure (DET). We denote by $R_{s}$ the set of all binary cell regions delineated by our cell segmentation method, while $R_{\text{Ref}}$ is the set of cell pixels from the reference region.

#### SEG measure –

It measures the Jaccard index between test and reference data, for each cell that has a reference mask. Then the method sets to zero the indices of cells for which $\left|R_{s}\cap R_{Ref}\right|\leq 0.5\cdot \left|R_{Ref}\right|$. It finally computes the average of all the individual indices to yield SEG.

#### DET measure –

DET calculates detection accuracy by comparing the nodes of the acyclic graphs generated by the tested algorithm with nodes of the acyclic graphs of the reference masks. It is defined as

(6) $$DET=1-min\left(AOGM_{D},AOGM_{D0}\right)/AOGM_{D0}.$$

Here $AOGM_{D}$ is the cost of transforming a set of nodes produced by the tested method into the set of reference nodes. $AOGM_{D0}$ is the cost of creating the set of reference nodes.

#### TRA measure –

It is based on comparison of acyclic oriented graphs produced by the tested and reference tracking methods. TRA is defined as a normalized Acyclic Oriented Graph Matching (AOGM) measure:

(7) $$TRA=1-min\left(AOGM,AOGM_{0}\right)/AOGM_{0},$$

where $AOGM_{0}$ is the AOGM value required for creating the reference graph from scratch.

### Results on CTC Training Data: Train on Sequence 1 and Test on Sequence 2

**Figure 4.**
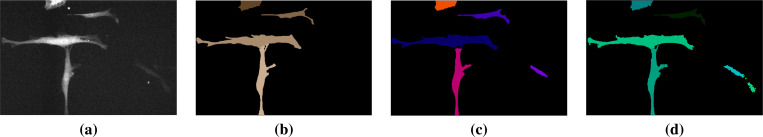
Segmentation comparisons for Fluo-C2DL-MSC-02: (a) Intensity frame, (b) GT reference segmentation, (c) ST reference segmentation, (d) Segmentation by the proposed method. This sequence contains overlapping elongated cells and has low temporal resolution. Our technique produced results that are notably close to the ST and GT references.

**Figure 5.**
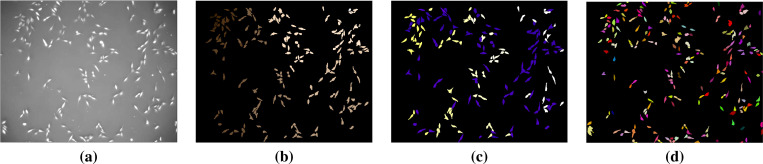
Segmentation comparisons for PhC-C2DL-PSC-02: (a) Intensity frame, (b) GT reference segmentation, (c) ST reference segmentation, (d) Segmentation by the proposed method. PSC has numerous small cells in each frame, with fine shape details, and several cell divisions occur over time. Our technique produced very good detection and tracking results.

**Figure 6.**
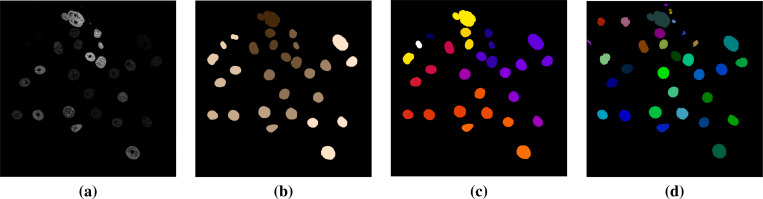
Segmentation comparisons for Fluo-N2DH-GOWT1-02: (a) Intensity frame, (b) GT reference segmentation, (c) ST reference segmentation, (d) Segmentation by the proposed method. This sequence is characterized by significant variability in contrast to noise ratios within each frame and over multiple time frames, that make cell detection difficult. The neural network classifier helped to detect cells of low contrast.

First, we present the results on CTC training data. In this experiment, we trained the foreground/background classifier on sequence 01 of each dataset and performed segmentation and tracking on sequence 02. Table 1 contains segmentation, detection and tracking measurements against GT and ST reference data. The dataset Fluo-N2DH-SIM+ has the same gold and silver truth labels because it is simulated. In addition, there is no ST reference data for detection and tracking, so we list the DET-GT and TRA-GT results only. From these results we observe general agreement among Jaccard and SEG for most of the datasets. A bit higher agreement is observed between DET and TRA measures. Overall, our technique yields the following average rates: Jaccard-ST: 0.741, SEG-ST: 0.785, SEG-GT: 0.729, DET-GT: 0.895, TRA-GT: 0.881. We note that the SEG-ST and SEG-GT rates are not directly comparable because ST contains dense annotations, but GT contains sparse annotations. We display segmentation label maps on a frame of three sequences in Figures 4, 5, and 6. We observe the good agreement between the cell label maps produced by our method and the reference label maps.

### Results on CTC Challenge Data

Next, we present results from our experiments on CTC challenge data. Our method’s code and results were submitted to CTC and re-produced by the challenge organizers for all participants. The organizers did not share reference data for these sequences. Hence, they calculated and posted evaluation performance measures of each method on the challenge website. Table 2 contains SEG, DET, and TRA results on each test sequence.

**Figure 7.**
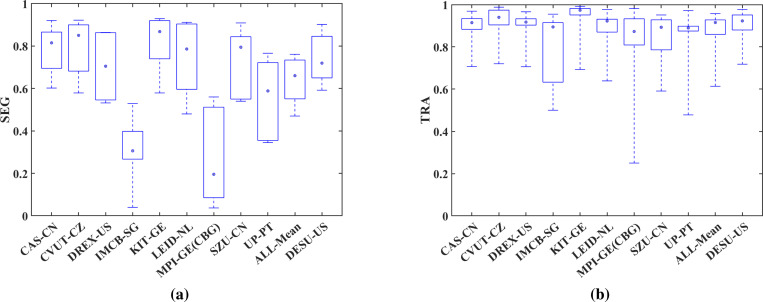
Performance evaluation of the six challenge datasets: (a) SEG measure comparisons, (b) TRA measure comparisons. Of note is the balanced performance of the proposed method over heterogeneous datasets that supports its generalizability.

Furthermore, in Tables 3 and 4 we list the SEG and TRA measures of other CTC participant techniques that were evaluated on the same test set with our technique. We also include the average performance measures of the 9 techniques, and our results for reference. Figure 7 shows boxplots of SEG and TRA values produced by the methods listed in Tables 3 and 4. Among the techniques that produced segmentation and tracking results for the same datasets, our technique ranked above the 70th percentile for 3 datasets in SEG and TRA values. Our technique scored mean SEG of 0.738 and mean TRA of 0.895 over all the tested sequences, ranking among the top 4 and top 3 techniques, respectively. It also achieves the top SEG and TRA performances among all compared methods for Fluo-C2DL-MSC.

## Discussion

By comparing the box plots of Figure 7 we observe that the proposed technique yields very good performances for all test datasets. These results indicate the generalizability of our technique to diverse datasets in terms of cell size and shape, density of environments, spatial and temporal scales, and contrast mechanisms. We summarize the main challenges presented by the utilized datasets next. The Fluo-C2DL-MSC sequences have overlapping elongated cells, with varying shapes and intensity profiles. These sequences have low spatial and temporal resolution. HeLa sequences present challenges for cell separation in a dense environment. PhC-C2DH-U373 has similar intensity levels inside the cell in cytoplasm and background with thick cell borders complicating cell boundary delineation. Fluo-N2DH-GOWT1 is characterized by significant variability in contrast to noise ratios within each frame and over multiple time frames, that make cell detection difficult. PhC-C2DL-PSC has numerous small cells in each frame, with fine shape details, and several cell divisions occur over time.

**Figure 8.**
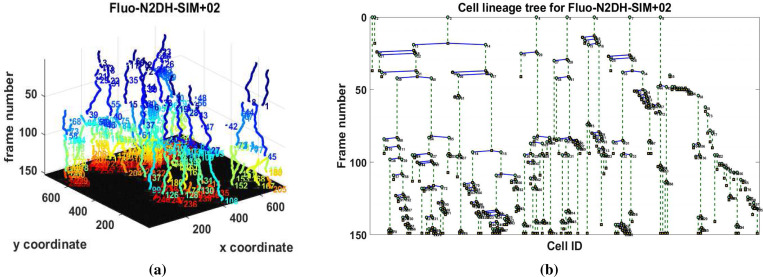
Visualization of cell tracking results for Fluo-N2DH-SIM+−02: (a) cell trajectories in 2D + time showing migration and division, (b) cell lineage tree showing cell events.

The high TRA rates show that our methodology can accurately create cell tracks and identify cell migration and cell mitosis. As seen in Figure 8, our method yields results that enable the monitoring and quantification of cell events in high throughput studies. These capabilities are significant for diagnosis of diseases such as cancer studies, drug development, and personalized medicine.

An original contribution of this work is the incorporation of multi-scale spatio-temporal features to perform automated scale selection, cell detection and segmentation. This approach helps to select a scale that automatically adjusts to the visual content of each frame, that addresses changes in contrast and noise levels, and reduces the need for hyperparameter tuning. Another key component of this approach is neural network-based detection of cell regions, and class prototype balancing using DBSCAN. DBSCAN is a non-parametric unsupervised learning technique that we use to identify the main cluster prototypes in the features to balance the class representations in neural network training. Our results showed that our neural network with DBSCAN-based re-sampling improves cell detection and segmentation compared to our previous approaches that employed parametric and nonparametric statistical tests ^40,41^.

In conclusion, we introduced a comprehensive and interpretable cell segmentation and tracking framework. Our extensive performance evaluation experiments showed its applicability to analysis of heterogeneous cell microscopy sequences. Future research directions include the development of a joint cell segmentation and classification approach, and graph neural network based optimization techniques for cell tracking and cell event detection.

## Figures and Tables

**Table 1.** Segmentation, detection and tracking performance measures on the second sequences of the CTC training datasets using the first sequences for training.

Dataset	Jaccard - ST	SEG - ST	SEG - GT	DET - GT	TRA - GT
Fluo-C2DL-MSC-02	0.7400	0.7769	0.6964	0.8128	0.7981
Fluo-N2DH-GOWT1-02	0.8551	0.9241	0.9252	0.9519	0.9519
Fluo-N2DL-HeLa-02	0.8382	0.8574	0.8052	0.9558	0.9480
PhC-C2DH-U373-02	0.6730	0.6948	0.7026	0.8457	0.8270
PhC-C2DL-PSC-02	0.6586	0.6729	0.6356	0.9172	0.8956
Fluo-N2DH-SIM+−02	0.6833	NO ST	0.6063	0.8844	0.8653

**Table 2.** SEG, DET, and TRA measures on CTC challenge datasets.

Dataset	SEG	SEG*μ*(±σ)	DET	DET*μ*(±σ)	TRA	TRA*μ*(±σ)

Fluo-C2DL-MSC-01	0.6734	0.650±	0.7099	0.735±	0.6895	0.718±
Fluo-C2DL-MSC-02	0.6273	0.033	0.7597	0.040	0.7456	0.035

Fluo-N2DH-GOWT1–01	0.8790	0.902±	0.9607	0.954±	0.9570	0.951±
Fluo-N2DH-GOWT1–02	0.9243	0.032	0.9470	0.008	0.9453	0.010

Fluo-N2DL-HeLa-01	0.8677	0.846±	0.9837	0.981±	0.9807	0.977±
Fluo-N2DL-HeLa-02	0.8236	0.031	0.9780	0.006	0.9724	0.004

PhC-C2DH-U373–01	0.7234	0.694±	0.9399	0.934±	0.9352	0.924±
PhC-C2DH-U373–02	0.6651	0.041	0.9281	0.015	0.9133	0.008

PhC-C2DL-PSC-01	0.5994	0.592±	0.9079	0.908±	0.8787	0.880±
PhC-C2DL-PSC-02	0.5841	0.011	0.9089	0.001	0.8805	0.001

Fluo-N2DH-SIM+−01	0.8562	0.745±	0.9812	0.932±	0.9777	0.922±
Fluo-N2DH-SIM+−02	0.6334	0.157	0.8828	0.079	0.8661	0.070

**Table 3.** SEG measure comparisons on all six challenge datasets.

Group	Fluo-C2DL-MSC	Fluo-N2DH-GOWT1	Fluo-N2DL-HeLa	PhC-C2DH-U373	PhC-C2DL-PSC	Fluo-N2DH-SIM+	Average

CAS-CN^42^	0.602	0.866	0.860	0.920	0.695	0.770	0.785
CVUT-CZ^20^	0.579	0.894	0.900	0.922	0.682	0.807	0.797
DREX-US^43^	0.546	0.864	0.863	0.679	0.532	0.731	0.702
IMCB-SG^44^	0.280	0.529	0.332	0.267	0.040	0.398	0.308
KIT-GE^21^	0.579	0.929	0.906	0.920	0.740	0.830	0.817
LEID-NL^13^	0.480	0.912	0.822	0.904	0.596	0.750	0.744
MPI-GE(CBG)^45^	0.101	0.085	0.290	0.037	0.512	0.560	0.264
SZU-CN^46^	0.540	0.909	0.844	0.832	0.550	0.757	0.731
UP-PT^47,48^	0.345	0.722	0.766	0.355	0.572	0.605	0.540

All-Mean	0.470	0.761	0.734	0.625	0.551	0.695	0.639

DESU-US	0.650	0.902	0.846	0.694	0.592	0.745	0.738

**Table 4.** TRA measure comparisons on all six challenge datasets

Group	Fluo-C2DL-MSC	Fluo-N2DH-GOWT1	Fluo-N2DL-HeLa	PhC-C2DH-U373	PhC-C2DL-PSC	Fluo-N2DH-SIM+	Average

CAS-CN^42^	0.707	0.883	0.898	0.969	0.931	0.934	0.887
CVUT-CZ^20^	0.720	0.904	0.988	0.974	0.925	0.955	0.911
DREX-US^43^	0.706	0.913	0.966	0.933	0.903	0.922	0.890
IMCB-SG^44^	0.633	0.882	0.915	0.955	0.500	0.907	0.799
KIT-GE^21^	0.693	0.951	0.992	0.982	0.968	0.979	0.927
LEID-NL^13^	0.639	0.92	0.931	0.977	0.870	0.925	0.877
MPI-GE(CBG)^45^	0.25	0.903	0.982	0.809	0.933	0.843	0.787
SZU-CN^46^	0.591	0.928	0.951	0.871	0.586	0.915	0.807
UP-PT^47,48^	0.478	0.875	0.972	0.883	0.898	0.896	0.834

All-Mean	0.613	0.911	0.957	0.928	0.859	0.920	0.865

DESU-US	0.718	0.951	0.977	0.924	0.880	0.922	0.895

## Data Availability

The research study has been conducted on open-access data publicly available at https://celltrackingchallenge.net
